# Global Patterns of Prostate Cancer Incidence, Aggressiveness, and Mortality in Men of African Descent

**DOI:** 10.1155/2013/560857

**Published:** 2013-02-13

**Authors:** Timothy R. Rebbeck, Susan S. Devesa, Bao-Li Chang, Clareann H. Bunker, Iona Cheng, Kathleen Cooney, Rosalind Eeles, Pedro Fernandez, Veda N. Giri, Serigne M. Gueye, Christopher A. Haiman, Brian E. Henderson, Chris F. Heyns, Jennifer J. Hu, Sue Ann Ingles, William Isaacs, Mohamed Jalloh, Esther M. John, Adam S. Kibel, LaCreis R. Kidd, Penelope Layne, Robin J. Leach, Christine Neslund-Dudas, Michael N. Okobia, Elaine A. Ostrander, Jong Y. Park, Alan L. Patrick, Catherine M. Phelan, Camille Ragin, Robin A. Roberts, Benjamin A. Rybicki, Janet L. Stanford, Sara Strom, Ian M. Thompson, John Witte, Jianfeng Xu, Edward Yeboah, Ann W. Hsing, Charnita M. Zeigler-Johnson

**Affiliations:** ^1^Department of Biostatistics and Epidemiology, Center for Clinical Epidemiology and Biostatistics, University of Pennsylvania School of Medicine, 217 Blockley Hall, 423 Guardian Drive, Philadelphia, PA 19104, USA; ^2^Abramson Cancer Center, University of Pennsylvania School of Medicine, Philadelphia, PA 19104, USA; ^3^Division of Cancer Epidemiology and Genetics, National Cancer Institute, Bethesda, MD 20892, USA; ^4^Department of Epidemiology, University of Pittsburgh, Pittsburgh, PA 15213, USA; ^5^Tobago Health Studies Office, Scarborough, Tobago, Trinidad and Tobago; ^6^Cancer Prevention Institute of California, Fremont, CA 94538, USA; ^7^Department of Health Research and Policy, Stanford University School of Medicine and Stanford Cancer Institute, Stanford, CA 94305, USA; ^8^Department of Medicine, University of Michigan Medical School, Ann Arbor, MI 48109, USA; ^9^The Institute of Cancer Research and Royal Marsden NHS Foundation Trust, Sutton, UK; ^10^Stellenbosch University and Tygerberg Hospital, Cape Town 7505, South Africa; ^11^Fox Chase Cancer Center, Philadelphia, PA 19111, USA; ^12^Hôpital Général de Grand Yoff, Université Cheikh Anta Diop de Dakar, Dakar, Senegal; ^13^Department of Preventive Medicine and Norris Comprehensive Cancer Center, University of Southern California, Los Angeles, CA 90033, USA; ^14^School of Medicine and Sylvester Cancer Center, University of Miami Miller, Miami, FL 33442, USA; ^15^The Johns Hopkins University School of Medicine and Bloomberg School of Public Health, Baltimore, MD 21287, USA; ^16^Department of Surgery, Brigham and Women's Hospital, Boston, MA 02138, USA; ^17^Department of Pharmacology and Toxicology, University of Louisville, Louisville, KY 40292, USA; ^18^Guyana Cancer Registry, Ministry of Health, Queenstown, Guyana; ^19^Department of Urology and the Cancer, Therapy and Research Center, The University of Texas Health Science Center at San Antonio, San Antonio, TX 78229, USA; ^20^Department of Public Health Sciences, Henry Ford Hospital, Detroit, MI 48202, USA; ^21^School of Medicine, University of Benin, Benin City, Nigeria; ^22^National Human Genome Research Institute, Bethesda, MD 20892, USA; ^23^Department of Cancer Epidemiology and Center for Equal Health, Moffitt Cancer Center, Tampa, FL 33612, USA; ^24^School of Clinical Medicine and Research, University of the West Indies, Nassau, Bahamas; ^25^Fred Hutchinson Cancer Research Center, Seattle, WA 98109, USA; ^26^Department of Epidemiology, MD Anderson Cancer Center, Houston, TX 77030, USA; ^27^Departments of Epidemiology and Biostatistics and Urology, Institute for Human Genetics, University of California, San Francisco, CA 94122, USA; ^28^Wake Forest University, Winston-Salem, NC 27157, USA; ^29^Korle Bu Teaching Hospital and University of Ghana Medical School, Accra, Ghana

## Abstract

Prostate cancer (CaP) is the leading cancer among men of African descent in the USA, Caribbean, and Sub-Saharan Africa (SSA). The estimated number of CaP deaths in SSA during 2008 was more than five times that among African Americans and is expected to double in Africa by 2030. We summarize publicly available CaP data and collected data from the men of African descent and Carcinoma of the Prostate (MADCaP) Consortium and the African Caribbean Cancer Consortium (AC3) to evaluate CaP incidence and mortality in men of African descent worldwide. CaP incidence and mortality are highest in men of African descent in the USA and the Caribbean. Tumor stage and grade were highest in SSA. We report a higher proportion of T1 stage prostate tumors in countries with greater percent gross domestic product spent on health care and physicians per 100,000 persons. We also observed that regions with a higher proportion of advanced tumors reported lower mortality rates. This finding suggests that CaP is underdiagnosed and/or underreported in SSA men. Nonetheless, CaP incidence and mortality represent a significant public health problem in men of African descent around the world.

## 1. Introduction

Little is known about the epidemiology of CaP among men in Sub-Saharan Africa (SSA) [1]. However, men of SSA descent around the world appear to suffer disproportionately from CaP compared to men of other races or ethnicities [2]. The International Agency for Research on Cancer (IARC) estimates that CaP is the leading cancer in terms of incidence and mortality in men from Africa and the Caribbean [3]. IARC also estimates that CaP is a growing problem in Africa with approximately 28,006 deaths from CaP in 2010, and approximately 57,048 deaths in 2030 [4]. This represents a 104% increase in the number of CaP deaths in Africa over the next two decades. However, CaP incidence and mortality rates that may be underestimated in SSA and possibly the Caribbean were compared to USA rates due to lack of screening, limited population-based cancer registry data, and underdiagnosis or treatment.

Comparisons of CaP incidence, prevalence, aggressiveness, and mortality in men of SSA descent are limited [5, 6]. Jackson et al. [7] reported that the age-adjusted incidence rate in a combined West African (Accra and Ibadan) series (36.7 per 1000) was almost equal to the rate in the Washington, DC series (40.6 per 1000). Although comparable prevalence data for Senegal are not available, Gueye et al. [8] compared clinical characteristics of CaP in African American (AA) and Senegalese men diagnosed between 1997 and 2002. Tumor stage was more advanced in Senegalese men than in AA men. These differences may reflect symptom-based diagnosis in Senegal compared to diagnosis following elevated PSA and/or positive digital rectal examination (DRE) in the USA. A number of papers in African populations have characterized PSA levels, use of screening, and the relationship of PSA with prostate tumor characteristics [9–13]. Similar differences between prostate cancer characteristics among men in New York, Guyana, and Trinidad have been reported with respect to tumor aggressiveness and survival [14]. These data suggest that important differences by geography exist among men of African descent and underscore the need for accurate CaP data for Caribbean, SSA, and AA populations in order to make meaningful comparisons. Insufficient population-based cancer registration in SSA continues to be a barrier to obtaining high quality data. 

## 2. Methods

### 2.1. Sources of Incidence and Mortality Rates

The primary data used for this report were derived from three sources. First, age-adjusted African-American rates were obtained for 2008 incidence from the 17 registry SEER data set (SEER-17) [15] and from 2008 mortality from national data [16] using SEER*Stat software [17]. National estimates of the number of cases were derived by multiplying the age-specific SEER-17 incidence rates by the corresponding national population estimates and summing over all age groups. For presentation of incidence time trends, we used the 13 registry SEER data set (SEER-13) [18] for the years 1992–2008. The USA data were tabulated using SEER*Stat software [17]. Second, estimated world incidence and mortality rates for the rest of the world were obtained from GLOBOCAN 2008 [4]. As with SEER data, these rates were adjusted according to the 1960 world standard. Third, a complete literature review was undertaken to identify studies reporting CaP rates in men of African descent by searching Medline for the keywords “prostate cancer”, “African”, “Caribbean”, and “Black.” Studies were initially not excluded based on sample size, study design, or methodological quality. To maximize comparability of studies across geography and/or time, studies were excluded if the ascertainment strategy or population base from which the estimates were derived could not be defined; estimates were based on hospital records, death certificates, or other data sources deemed to be potentially unreliable, or if rates were not age-standardized to the 1960 world population. All rates presented here were age-standardized to the 1960 world population to be comparable with other reports of cancer incidence. Data for Africa and the Caribbean from GLOBOCAN 2008 reflect the total population of the country or region being reported, and therefore include men who are of non-African descent. The proportion of men of non-African descent in each country or region is shown in Supplementary Table 1 available online at http://dx.doi.org/10.1155/2013/560857 if known. To limit the inferences to countries where the majority of the population is of African descent, we excluded countries where <50% of individuals were reported to be of African descent (e.g., Puerto Rico and Cuba).

### 2.2. Studies and Consortia

The men of African descent and Carcinoma of the Prostate (MADCaP) consortium and the African Caribbean Cancer Consortium (AC3) contributed data to this report. The goal of these consortia is to develop multicenter studies that address CaP research at a variety of levels in men of SSA descent in North America, the Caribbean, Europe, and Africa. MADCaP and AC3 studies include epidemiological, biomarker, genetic, risk assessment, and outcomes prediction research. Data regarding prostate tumor characteristics were collected from the following studies: the CAPGenes Prostate Cancer Genetics Studies (CAPGenes) [19], Fred Hutchinson Cancer Research Center (FHCRC) CaP Studies [20, 21], the Prostate Cancer Risk Assessment Program (PRAP) at Fox Chase Cancer Center [22], the Flint Men's Health Study (FMHS) [23, 24], Gene-Environment Interaction in CaP (GECAP) Study at Henry Ford Hospital [25], Los Angeles County Study (LACS) [26], CaP Clinical Outcome Study (PC^2^OS) at the University of Louisville [27], MD Anderson Cancer Center [28], the Multiethnic Cohort Study (MEC) [29], Moffitt Cancer Center Study [30], NCI Prostate Tissue Study (NCIPTS), University of Pennsylvania Study of Cancer Outcomes, Risk, and Ethnicity (SCORE) [31], University of Texas San Antonio Center for Biomarkers of Risk for CaP (SABOR), University of Texas Health Science Center at San Antonio [32, 33], San Francisco Bay Area Prostate Cancer Study (SFBAPCS) [34], United Kingdom Genetic CaP Study (UKGPCS), and the Wake University Consortium including participants from the Johns Hopkins University, Wake Forest University, and Washington University (St. Louis) [35], the PROGRES (Prostate Genetique Recherche Senegal) study in Dakar, Senegal [8, 36], the Ghana Prostate Health Study (GPHS) in Accra, Ghana [37], the Tygerberg Hospital study in Cape Town, South Africa [38], the Guyana Tumor Registry [14], the Tobago Prostate Cancer Screening Study [39], and unpublished data from the Bahamas Prostate Cancer Study. At all MADCaP and AC3 centers, data were collected under protocols approved by local IRBs.

### 2.3. Assessment of CaP Ascertainment

Correlations between percent T1 stage tumors and CaP incidence, mortality, and population statistics on medical care were computed using Spearman rank correlations, using abstracted literature review and tumor stage data from the MADCaP consortium. Sources of percent of gross domestic product spent on health care outside the USA were obtained from WHO World Health Statistics 2010 [42]. Comparable data for the USA were obtained from the Centers for Medicare and Medicaid Services by state [43]. STATA 10 was used for all descriptive and statistical analyses of prostate tumor characteristics, incidence, and mortality trends. All *P* values are based on two-sided tests at *α* = 0.05. Statistically significant differences from the null hypothesis were inferred at *P* < 0.05.

## 3. Results

### 3.1. Recent Estimates of International Cancer Incidence and Mortality

We estimated that more than 30,000 cases of CaP were diagnosed among AA men in 2008 (Table 1). The GLOBOCAN estimate for men in all of Africa in 2008 was more than 39,000 CaP cases; the number in SSA would be smaller, but was not estimated. The estimated number among Caribbean men was half of the number in the USA or Africa. CaP was by far the most frequently diagnosed reportable cancer during 2008 in AA, Caribbean, and SSA men, with the age-standardized CaP incidence rates ranging from 159.6 per 100,000 among AA to 71.1 in the Caribbean and 17.5 in Africa. The 28,000 deaths due to CaP in Africa were more than five times the 4,600 among AA and four times the 6,500 among Caribbean men. The mortality rate for CaP exceeded that for any other cancer in Africa (12.5 per 100,000) and the Caribbean (26.3 per 100,000), and followed only lung cancer among AA (22.4 versus 49.1 per 100,000). Whereas the CaP incidence rate was highest among AA men, the CaP mortality rate was highest in Caribbean men. Both the CaP incidence and mortality rates were lower in Africa than in AA or Caribbean men. As a result, the mortality : incidence rate ratios ranged from 0.71 in Africa to 0.41 in the Caribbean and 0.14 in AA. 

### 3.2. International Variation in CaP Incidence

**Figure 1:**
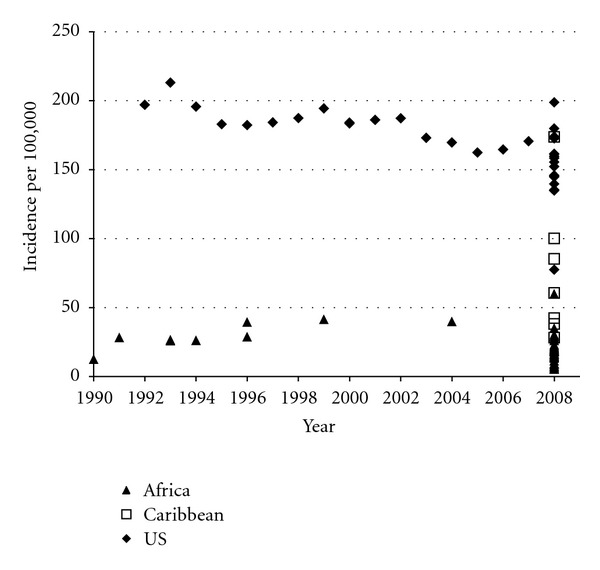
Estimates of CaP incidence (1990–2008) per 100,000 men of African descent, age-standardized to the 1960 world Population. Incidence rates for the USA are from SEER-13 for 1992–2008; data for 2008 include one estimate from each of the SEER-17 registries, except two registries with <16 cases. Incidence rates for all Caribbean countries are from 2008 GLOBOCAN data. Incidence rates for African countries are from specific population-based cancer registries for 1990–2004 and from 2008 GLOBOCAN data. Rates can be found in Supplementary Table 1. Incidence rates are age-standardized to the 1960 world population.

Figure 1 presents the age-standardized CaP incidence rates in men of African descent from SSA, the Caribbean, and the USA SEER program for various years 1990–2008. Supplementary Table 1 presents the incidence rates and references for the information plotted in Figure 1. Supplementary Table 2 presents rates that were not included in Figure 1 because they represent estimates that were not standardized to the 1960 world population, did not reflect a clearly defined reference population, were hospital or clinic-based, or for which the sampling or ascertainment frame was not clearly defined. Many of these rates vary widely, suggesting that they may not be accurate. As shown in Figure 1 (and Supplementary Table 1), incidence rates in Africa historically have been <50 per 100,000, based on data from Uganda and Zimbabwe. Rates in SEER-13 and SEER-17 cancer registries have all exceeded 150 per 100,000, with a pronounced peak at 213.1 during 1993 after the introduction of widespread and adoption of PSA testing and nadir at 163.4 during 2005.

During 2008, CaP incidence internationally was highest in the USA Atlanta SEER registry (198.8 per 100,000) and lowest in Niger (5.1 per 100,000), with substantial variability by geography. Within the SEER-17 cancer registries, after excluding two registries with <16 cases, rates ranged from 134.8 per 100,000 in Iowa to 198.8 per 100,000 in Atlanta. In the Caribbean, rates varied from 51.1 per 100,000 in Jamaica to 173.7 per 100,000 in Martinique. In SSA, incidence rates were generally lower than those in the USA or Caribbean varying from 5.1 per 100,000 in Niger to 59.7 per 100,000 in South Africa.

### 3.3. International Variation in CaP Aggressiveness

Table 2 reports characteristics of prostate tumors in Africa, the Caribbean, the UK, and the USA based on literature review and data from the MADCaP Consortium. These data represent clinical data from observational (e.g., cohort, case-control, or population-based screening) studies, and therefore may not represent the distribution of traits in the population as a whole. In addition, some studies have preferentially ascertained advanced disease, hospital-based cases, or population-based cases. Some imposed age restrictions on case ascertainment. Therefore, the proportions reported here reflect not only geographic differences but also differences in study design. The proportion of tumors that were T1 stage in AA men ranged from 35–63%, compared with 15–29% in SSA, and 24% in the Caribbean and 34% in the UK. There were significant differences in Gleason score (*P* = 0.003), tumor stage (*P* < 0.001), and PSA levels at diagnosis (*P* < 0.0001) between USA, UK, and African studies. A significantly greater proportion of tumors in Africa had a high Gleason score or high tumor stage compared with those in the USA or UK. In the UK and USA, the most common Gleason scores are 6 and 7, whereas the distribution in other regions is more uniform, with lower (≤5) and higher (≥8) scores representing a greater proportion of tumors than in the USA. The tumor stage distribution differed substantially by geography, with a larger proportion of stage T3/T4 tumors in Africa than other locations. PSA levels at diagnosis were lowest in the USA and Tobago. There were no statistically significant differences in Gleason score (*P* = 0.07), tumor stage (*P* = 0.51), or PSA at diagnosis (*P* = 0.24) among centers within Africa. 

### 3.4. International Variation in CaP-Specific Mortality

**Figure 2:**
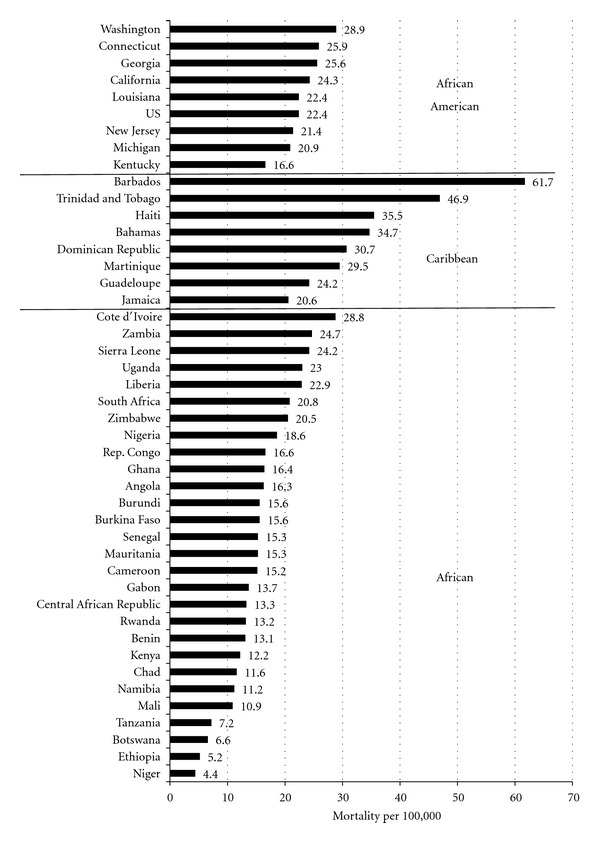
Estimates of 2008 CaP mortality per 100,000 men, age-standardized to the 1960 world population. African American data are from states with at least one SEER-17 cancer registry (excluding states with fewer than 10 CaP deaths), African and Caribbean data are from GLOBOCAN 2008.

Table 1 suggested that the CaP ratio of mortality to incidence in Africa (0.71) is notably higher than that in the Caribbean (0.41) or AA men (0.14). To further explore mortality rates in these regions, Figure 2 presents CaP mortality rates (standardized to the 1960 world population) in men of African descent in the USA, SSA, and the Caribbean. The mortality estimates vary considerably and overlap across regions. In the Caribbean, CaP mortality rates were comparable to or higher than in AA men. For example, the total AA mortality rate was 22.4 per 100,000 compared with estimates as high as 61.7 per 100,000 in Barbados [44], which was the highest reported in the world. In SSA, estimated mortality rates were generally lower than those in the USA or Caribbean. Mortality estimates in 2008 ranged from a low of 4.4 per 100,000 in Niger to 28.8 per 100,000 in Cote d'Ivoire [44]. Indeed some rates in AA were lower than several in Africa and many in the Caribbean. As shown in Table 2, the proportion with high stage/grade disease at diagnosis is much greater in Africa than in AA. 

### 3.5. Completeness of CaP Ascertainment

**Figure 3:**
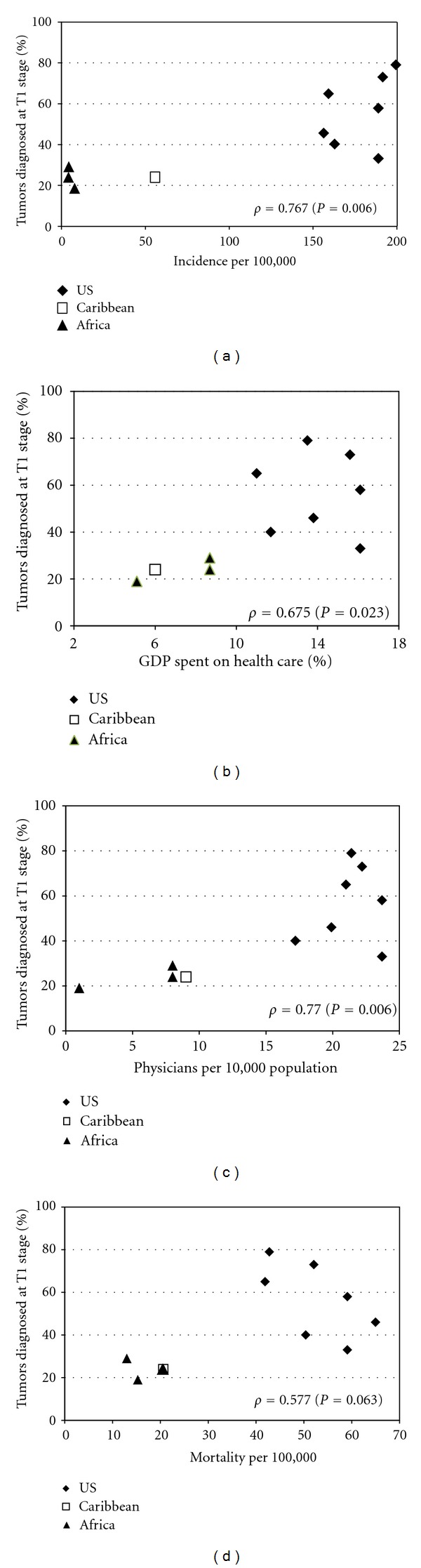
Relationship of prostate tumor aggressiveness with CaP incidence, mortality, and health care-related statistics in men of African descent. % T1 stage tumor data from the MADCaP and AC3 Consortia. (a) Percent of tumors diagnosed at T1 stage by PCa incidence (per 100,000 population). Age-standardized incidence rates from 2008 GLOBOCAN and 2008 SEER-17 cancer registries (San Francisco-Oakland, San Jose/Monterey, and Los Angeles, California; Connecticut; Detroit; Louisiana; New Jersey). (b) Percent of tumors diagnosed at T1 stage by percent of gross domestic product spent on health Care. Sources of percent of gross domestic product spent on health care: (1) Outside USA: WHO World Health Statistics 2010 (http://www.who.int/whosis/whostat/EN_WHS10_Full.pdf); (2) USA: Centers for Medicare and Medicaid Services (https://www.cms.gov) by state. (c) Percent of tumors diagnosed at T1 stage by number of physicians per 10,000 persons. Sources of data on physicians per 10,000 persons: (1) Outside USA: WHO World Health Statistics 2010 (http://www.who.int/whosis/whostat/EN_WHS10_Full.pdf); (2) USA (by state): Dionne, M., Moore, J., Armstrong, D., Martiniano, R. (2006) The United States health workforce profile. Rensselaer, NY: Center for Health Workforce Studies, School of Public Health, SUNY Albany. (d) percent of tumors diagnosed at T1 stage by CaP mortality (per 100,000 population). Mortality data for 2006 from CDC Wonder Database (http://wonder.cdc.gov/).

Figure 3 presents the relationship of the proportion of tumors diagnosed at T1 stage, based on data from the MADCAP consortium, with characteristics that may be related to CaP detection: CaP incidence, based on cancer registry data (Figure 3(a)), percent of gross domestic national or state product spent on health care (Figure 3(b)), and national or state number of physicians per 10,000 persons (Figure 3(c)). As shown in Figures 3(a)–3(c), there was a strong positive correlation between each of these variables and percent of tumors diagnosed at T1 stage. Figure 3(d) also presents the relationship of T1 stage tumors by CaP mortality in SSA, the Caribbean, and several USA states for which T1 stage data were available. Figure 3(d) suggests that the higher the proportion of T1 stage tumors diagnosed, the higher the mortality rate. Stated differently, mortality rates are lower in SSA and the Caribbean, where T1 stage tumors are least likely to be diagnosed. However, given that the mortality : incidence rate ratio (Table 1) of cancers in Africa (0.71) and the Caribbean (0.41) are substantially higher than in the USA (0.14), these results suggest that there is a substantial underreporting of cases of CaP in at least some parts of SSA and the Caribbean. 

## 4. Discussion

Our data suggest that among men of Africa descent, CaP is most common in AA and Caribbean men, and considerably less common in SSA (Figure 1). These data also suggest that CaP is a major cancer in men of African descent throughout the world, and that the currently available incidence and mortality rates may represent an underestimate of the actual CaP incidence and mortality rates in SSA and the Caribbean. Despite the evidence that CaP occurs at high rates in AA men, relatively little data are available regarding the epidemiology of CaP in men of African descent in other locations. Possible explanations for the wide range in CaP incidence and mortality by geography observed here fall into several categories: (1) differences in health care access, diagnosis, and screening; (2) differences in the methodology used to generate rates including completeness of ascertainment and (3) underlying differences in risk due to demographic differences, genetics/biology, lifestyle, or environmental exposures.

### 4.1. Health Care Access and Screening

PSA is widely used in the USA for detection of CaP. While PSA improves CaP detection beyond digital rectal examination (DRE) [45], its sensitivity and specificity are suboptimal, and it has not been widely implemented in clinical practice in many parts of the world. PSA testing is responsible for a sharp peak in the incidence of prostate tumors detected in the USA and other countries between 1990–1995, but the additional cases detected during this period were primarily localized disease [46]. Many of these tumors may not have been diagnosed until later, if at all, or until they caused clinically relevant events had PSA testing not been in use [47]. Data regarding screening practices and rates, as well as time trends in mortality, are not widely available for African descent populations outside the USA. 

The use of PSA testing impacts the international comparisons made here. While the prevalence of PSA testing is not well documented, it appears that CaP is also commonly detected when PSA testing is performed in Caribbean men [39]. No data exist regarding the prevalence of PSA usage in Africa, but it is believed that PSA testing is not common. Thus, lower rates of CaP in Africa may in part reflect lower probability of CaP detection than in countries like the USA where PSA and DRE screening are more widely used. Compounding the lower use of PSA testing is issues related to access to cancer-related health care, including diagnosis and treatment of disease. There are currently no data on the proportion of CaP that is never diagnosed in a clinical setting in Africa. 

### 4.2. Methodological Issues in Estimating CaP Incidence and Mortality

Limitations in the availability of data in some regions limit our ability to make strong inferences about the actual rates of CaP in some countries and to make valid comparisons of these rates across countries. Direct comparisons of rates globally are limited by a number of methodological issues. Different age standardizations may be used in different studies. All data presented here present age-standardized rates standardized to the 1960 world population to maximize comparability. Limitations in available data also preclude strong inferences and comparisons of international rates. In many countries, incidence and mortality rates do not separate out men of African descent from men of other ethnicities. For example, the available data in the Caribbean and Africa represent all men, regardless of race. Thus, the rates of CaP reported here are meant to reflect those in men of African descent, but in fact they represent the rates for all men in these countries (aside from the countries we have omitted where the proportion of African descent men was low). We have presented data obtained from population-based cancer registries (Supplementary Table 1), but many reports rely on data obtained from hospital-based or other ascertainment strategies that may not reflect CaP cases in the general population (Supplementary Table 2), or may comprise a biased subset of cases. Thus, some reports suggest very low or very high rates of CaP, but it is not clear whether these reports are valid and they may not be useful in making international comparisons. We have also not presented direct comparisons of rates across populations because data were not available to obtain meaningful variance estimates around incidence or mortality rates across populations. However, to the degree that the rates reflect the correct population estimates based on large samples, *P* values may not be required to compare relative rates across populations. Similarly, reports of mortality in Africa are captured by various means, and the accuracy of cause of death reports in addition to completeness of reporting may be inadequate. Therefore, comparisons of USA, Caribbean, and SSA rates may be limited by differences in data reporting across regions.

### 4.3. Underlying Risk Profile Differences

At least two hypotheses can be proposed to explain differences in CaP risk across populations of African descent. First, it is possible that CaP is underdiagnosed in the less developed world, particularly in SSA, and the rates are in fact higher than is suggested by existing data due to limited tumor detection. This hypothesis is addressed below. Second, it is possible that CaP incidence is truly lower in the developing world than in the developed world. This might imply that SSA populations are less susceptible to CaP due to genetic risk, environmental exposures, or other risk factors that cause CaP. Differences in life expectancy in developed countries versus Africa may influence CaP rates. Differences in underlying risk profiles across populations may also explain in part differences in risk across populations. Recent studies have identified a large number of loci that are involved in CaP susceptibility, and there is growing evidence that the frequency and contribution of these risk alleles to CaP differ across populations. Chang et al. [48] reported the multicenter MADCaP study of CaP susceptibility using a sample of nearly 8 000 men of African descent in the USA and UK. They reported that the majority of the loci identified as CaP susceptibility loci in White or Asian populations were not replicated in men of African descent. Haiman et al. [49] reported a genome-wide association study (GWAS) using a sample of over 6 000 African descent CaP cases and controls that overlapped with the sample of Chang. This GWAS also did not replicate most of the previously reported loci identified in European or Asian descent populations [50]. Haiman et al. [49] also identified a novel locus on chromosome 17 that had not been identified in prior GWAS. The risk variant at this new locus was only identified in African descent populations and was not present in European descent populations. Explanations for the lack of replication across racial groups include the possibility that the underlying genetic etiology of CaP differs across racial groups, and that the frequency of risk alleles in different populations is sufficiently variable that the effects of these variants cannot be detected in some groups. Finally, it is possible that genetic effects are dependent on nongenetic exposures, such that the main effect of a locus differs depending on the environmental context in which it is acting. Given the major differences in environmental exposures, including infection and lifestyle across populations, this is a likely hypothesis that requires additional study.

Even though environment can be hypothesized to play a major role in CaP risk, there have been few consistent exposures identified to date that are clearly associated with CaP risk. The only epidemiological factors that are uncontested are family history of prostate cancer, age, and race. Therefore, it cannot be clearly determined that environmental exposure differences across populations explain different CaP rates given that the role of these exposures on risk remains unclear.

### 4.4. Is There Evidence for Underascertainment of CaP in Some Populations?

Our data suggest that CaP rates may be underestimated in SSA and possibly the Caribbean. This underascertainment may be due to CaP underdiagnosis, under-ascertainment, or both. It is not possible using the data available here to distinguish these possible explanations for a potential underestimation of CaP. We evaluated the relationship of early stage tumors (i.e., stage T1) with characteristics of the populations in which CaP is diagnosed (Figure 3). Early stage tumors were strongly correlated with higher incidence, greater percent of gross domestic product spent on health care, and greater number of physicians per population. While certainly not conclusive, these data support the hypothesis that countries where greater medical intervention and access to health care are available have higher rates of early stage prostate tumors. This finding is not surprising but is consistent with the hypothesis that CaP incidence rates in SSA are underestimated. However, it is critical to remember that small numbers of cases, usually obtained from clinic- or hospital-based series, may reflect relatively less stable or representative information than those obtained from population-based cancer registries (e.g., SEER) in the developed world.

Evaluation of the variability in CaP rates is severely limited by the availability of appropriate cancer registries in SSA. According to IARC, approximately 38 cancer registries were active in SSA in 2011, but only two of these were fully compliant with IARC standards. One reason often cited for the lack of compliance with IARC standards is that registries may not have access to all newly diagnosed cancer cases in the population and cannot provide pathology confirmation of diagnoses due to limited pathology resources in many countries. Evaluation of the accuracy of cancer incidence rates obtained from population-based cancer registries in Africa has been assessed in Uganda [51]. The evidence from that assessment suggests accurate cancer rates can be obtained in Africa if standardized protocols are followed. Thus, additional population-based cancer registries should be developed using accepted protocols in SSA to obtain accurate estimates of cancer incidence and mortality.

In addition to limitations in the estimates of incidence and mortality from population registries, it is not currently possible to make global comparisons of differences among these rates using available methods. While it is possible to estimate variances and standard errors around the rates of interest when observed counts are available (e.g., SEER data), the standard in the field is to not estimate variances for GLOBOCAN type estimates because they are based around estimated counts, not actual observed counts. While formulae exist that could be used to estimate variants, these are unlikely to be accurate because there are many sources of uncertainty and many assumptions made in the GLOBOCAN estimation process. Ferlay et al. [52] report that the some necessary assumptions for variance estimation could be quantified, whereas for others they clearly could not be. Thus, it is not possible with current methodology to obtain a single variance estimate that is appropriate for data from all locations. Since it is not acceptable to make comparisons of rates across different locations based around inaccurate variance estimates, we do not estimate variances, nor do we make direct comparisons of differences in rates across populations. 

Although we advocate for improved reporting of cancer data to facilitate research and clinical practice, we are aware that there have been concerns about the overdiagnosis of CaP. Therefore, we do not advocate increased use of PSA screening in SSA, but to develop appropriate approaches to CaP screening in SSA that will limit CaP-related morbidity and mortality.

## 5. Conclusions

CaP is estimated to be the most common tumor in SSA and Caribbean men. However, accurate estimates of CaP incidence and mortality, particularly in SSA, do not adequately inform appropriate CaP screening and clinical practice. The data presented here do not provide specific guidance about optimal prostate cancer screening or treatment strategies in Africa. However, it is likely that African-specific risk profiles, disease aggressiveness, and health care access mean that screening and treatment strategies may be different in Africa than those in standard practice in North America or Europe. If CaP is actually more common than is currently understood in African descent populations, then CaP incidence rates are likely to be higher and may represent an even greater public health problem than is currently thought. Additional attention to the problem of CaP in Africa is warranted that could include development of epidemiological research and resources (e.g., high quality population-based cancer registries) to improve our understanding of the CaP burden in African populations; epidemiological and genetics research to understand the risk factors acting in Africa; studies to determine appropriate use of PSA testing; and treatment-related research that will lead to reduced CaP morbidity and mortality in the Caribbean and SSA.

## Supplementary Material

Supplementary Table 1 presents the incidence rates and references for the information plotted in Figure 1. Supplementary Table 2 presents rates that were not included in Figure 1 because they represent estimates that were not standardized to the 1960 world population, did not reflect a clearly defined reference population, were hospital or clinic-based, or for which the sampling or ascertainment frame was not clearly defined.

## Figures and Tables

**Table 1:** Age-Standardized Estimates (ASE) of incidence and mortality during 2008 for five leading cancers in men of African descent by Geography.

Location	Incidence*			Mortality*			Prostate Cancer Mortality : incidence rate ratio
	Cancer	Number of cases	ASE**	Cancer	Number of deaths	ASE**	
Africa	*Prostate *	**39,460**	**17.5**	*Prostate *	**28,006**	**12.5**	**0.71**
	Liver	34,612	11.7	Liver	33,826	11.7	
	Lung	20,821	8.4	Lung	19,429	7.9	
	Colorectal	19,049	6.9	Esophagus	16,678	6.5	
	Bladder	16,938	6.7	Colorectal	14,707	5.5	

Caribbean	*Prostate *	**15,950**	**71.1**	*Prostate *	**6,543**	**26.3**	**0.41**
	Lung	5,555	25.7	Lung	5,157	23.6	
	Colorectal	3,186	14.4	Colorectal	2,010	8.8	
	Stomach	2,418	11.2	Stomach	1,769	8.0	
	Larynx	1,469	7.1	Liver	1,304	6.1	

US (African American)	*Prostate *	**30,068**	**159.6**	Lung	9,629	49.1	
	Lung	11,712	60.4	*Prostate *	**4,587**	**22.4**	**0.14**
	Colorectal	8,298	42.0	Colorectal	3,478	17.5	
	Kidney	3,600	18.2	Pancreas	1,894	9.6	
	Bladder	2,415	12.2	Liver	1,748	8.7	

*African and Caribbean incidence and mortality estimates from GLOBOCAN 2008 and include men of all races. US incidence rates from SEER-17, estimated numbers of cases for the total US based on the SEER-17 rates, and US mortality data for the entire country; all include only African American men.

**ASE: Age-Standardized Estimates per 100,000 population adjusted to the 1960 world population.

**Table 2:** International variation in prostate tumor characteristics in men of African descent.

Region	Location	Data source*	*N*	Mean age at diagnosis (Yrs, range)	Median PSA (ng/mL, range)	Gleason score, %					Tumor stage, %			
						≤5	6	7	8	9+	T1	T2	T3	T4
Africa	Dakar, Senegal	HBCC	114	68 (41–95)	59.5 (0.5–6,190)	39	18	19	20	4	19	35	30	16
	Accra, Ghana	PSS	689	69 (42–95)	52.0 (0.7–8,423)	68	12	8	6	6	15	42	21	22
	Cape Town, South Africa: Coloured	HBCC	207	67 (46–94)	19.3 (0.5–14,390)	20	31	21	12	16	29	30	19	22
	Cape Town, South Africa: Black	HBCC	23	70 (52–90)	37.2 (5–3,308)	22	11	11	33	22	25	20	25	30

Caribbean	Guyana	HBCS	169	74 (26–98)	NA	NA	NA	NA	NA	NA	91 (T1 + T2)		4	5
	Jamaica [40]	HBCS	529	71 (41–91)	30.7 (12–109)	1	37	32	19	11	NA	NA	NA	NA
	Jamaica [41]	HBCS	99	72 (50–90)	37.0 (1–2,100)	0	16	24	41	18	24	39	21	9
	Tobago	PBR	508	65 (40–79)	6.3 (0.3–18,330)	.5	46	41	6	6	NA	NA	NA	NA

UK	Greater London	HBCS	177	71 (48–87)	107.6 (1–2,463)	10	40	38	9	3	34	42	20	4

US	Northeast**	HBCC	879	59 (36–88)	5.9 (0.3–69)	2	51	40	5	3	38	35	27	0.6
	Southeast**	HBCC	727	61 (91.36)	6.4 (0–5,000)	8	36	37	8	10	35	51	13	0.6
	Midwest**	HBCC	533	69 (38–97)	7.0 (0–14,635)	2	24	54	10	10	63	32	5	0
	West**	PBCC, PCS, HRCC	1,474	74 (42–91)	NA	7	30	40	15	7	89 (T1 + T2)		10	0.5

*Study type: HBCC: hospital-based case-control; HBCS: hospital-based case series; HRCC: high risk (aggressive disease) case-control study; PBCC: population-based case-control; PBR: population-based cancer registry; PCS: prospective cohort study; PSS: population screening study.

**MADCaP Groups: Northeast: Philadelphia, Baltimore, Washington DC; Southeast: Louisville, Houston, San Antonio, Tampa, Wake Forest; Midwest: Cleveland, Detroit, Flint, St. Louis; West: Seattle, Los Angeles, San Francisco Bay Area. Data from other centers as described in methods; citations indicate data were taken from the literature only; NA: Not Available.
