# Supplementary material for: Global Patterns of Prostate Cancer Incidence, Aggressiveness, and Mortality in Men of African Descent
Source: Prostate Cancer. 2013 Feb 13;2013:560857. doi: 10.1155/2013/560857 (PMC3583061; doi:10.1155/2013/560857)
Supplement: Supplementary file 1 — Supplementary Table 1 presents the incidence rates and references for the information plotted in Figure 1. Supplementary Table 2 presents rates that were not included in Figure 1 because they represent estimates that were not standardized to the 1960 world population, did not reflect a clearly defined reference population, were hospital or clinic-based, or for which the sampling or ascertainment frame was not clearly defined. [file 560857.f1.pdf]

Supplementary Table 1: CaP Incidence Data in Men of African Descent Reported in Figures. All data are age-standardized to the 1960 world population.

Region	Location	Year(s)	Age-Standardized Incidence per 100,000	Source	Population of African Descent (%)[a]	Reference
Africa	Angola	2008	19.7	GLOBOCAN	99	[4]
	Benin	2008	16.0	GLOBOCAN	99	[4]
	Botswana	2008	8.9	GLOBOCAN	NA	[4]
	Burkina Faso	2008	18.3	GLOBOCAN	NA	[4]
	Burundi	2008	18.6	GLOBOCAN	>99	[4]
	Cameroon	2008	19.2	GLOBOCAN	>99	[4]
	Cape Verde	2008	18.8	GLOBOCAN	99	[4]
	Central African Republic	2008	16.3	GLOBOCAN	>98	[4]
	Chad	2008	14.0	GLOBOCAN	>94	[4]
	D.R. Congo	2008	14.3	GLOBOCAN	NA	[4]
	Rep. Congo	2008	21.0	GLOBOCAN	97	[4]
	Cote d'Ivoire	2008	34.8	GLOBOCAN	97	[4]
	Djibouti	2008	7.1	GLOBOCAN	>95	[4]
	Equatorial Guinea	2008	18.9	GLOBOCAN	>98	[4]
	Eritrea	2008	7.8	GLOBOCAN	>99	[4]
	Ethiopia	2008	6.3	GLOBOCAN	>89	[4]
	Gabon	2008	18.2	GLOBOCAN	NA	[4]
	Ghana	2008	20.4	GLOBOCAN	>92	[4]
	Guinea	2008	20.1	GLOBOCAN	NA	[4]
	Guinea-Bissau	2008	17.9	GLOBOCAN	>99	[4]
	Kenya	2008	15.2	GLOBOCAN	99	[4]
	Lesotho	2008	11.1	GLOBOCAN	>99	[4]
	Liberia	2008	27.5	GLOBOCAN	NA	[4]
	Madagascar	2008	21.8	GLOBOCAN	NA	[4]
	Malawi	2008	13.5	GLOBOCAN	>97	[4]
	Mali	2008	13.1	GLOBOCAN	>95	[4]
	Mauritania	2008	17.9	GLOBOCAN	NA	[4]
	Mozambique	2008	16.1	GLOBOCAN	>99	[4]
	Namibia	2008	14.8	GLOBOCAN	94	[4]
	Niger	2008	5.1	GLOBOCAN	>99	[4]
	Nigeria	2008	22.7	GLOBOCAN	NA	[4]
	Rwanda	2008	16.2	GLOBOCAN	NA	[4]
	Senegal	2008	18.1	GLOBOCAN	99	[4]
	Sierra Leone	2008	27.3	GLOBOCAN	NA	[4]
	Somalia	2008	8.9	GLOBOCAN	>99	[4]
	South Africa	2008	59.7	GLOBOCAN	88	[4]
	Sudan	2008	9.0	GLOBOCAN	>97	[4]
	Swaziland	2008	17.6	GLOBOCAN	97	[4]
	Tanzania	2008	8.8	GLOBOCAN	99	[4]
	The Gambia	2008	5.4	GLOBOCAN	99	[4]
	Togo	2008	28.8	GLOBOCAN	>99	[4]
	Uganda	2008	28.7	GLOBOCAN	NA	[4]
	Uganda (Kampala)	1989-1991	12.3	Registry	NA	[53]

Region	Location	Year(s)	Age-Standardized Incidence per 100,000	Source	Population of African Descent (%)[a]	Reference
	Uganda (Kampala)	1991-1995	25.7	Registry	NA	[54]
	Uganda (Kampala)	1996-2001	41.2	Registry	NA	[54]
	Uganda (Kampala)	2002-2006	39.6	Registry	NA	[54]
	Uganda (Kyadondo)	1991-1994	26.3	Registry	NA	[55]
	Uganda (Kyadondo)	1995-1997	39.2	Registry	NA	[55]
	Zambia	2008	30.0	GLOBOCAN	>99	[42]
	Zimbabwe	2008	26.0	GLOBOCAN	>98	[42]
	Zimbabwe - Harare (African)	1990-1992	28.1	Registry	>98	[56]
	Zimbabwe - Harare (African)	1993-1995	26.0	Registry	>98	[56]
	Zimbabwe - Harare (African)	1994-1997	28.5	Registry	>98	[57]
Caribbean	Bahamas	2008	78.5	GLOBOCAN	85	[4]
	Barbados	2008	140.0	GLOBOCAN	96	[4]
	Dominican Republic	2008	68.8	GLOBOCAN	84	[4]
	Guadeloupe	2008	108.2	GLOBOCAN	NA	[4]
	Haiti	2008	78.4	GLOBOCAN	>95	[4]
	Jamaica	2008	51.1	GLOBOCAN	>97	[4]
	Martinique	2008	173.7	GLOBOCAN	NA	[4]
	Trinidad and Tobago	2008	89.4	GLOBOCAN	>58	[4]
US	Atlanta	2008	198.8	SEER-17	b	[15, 58]
	California ex. SF/SJ/LA	2008	145.2	SEER-17	b	[15, 58]
	Connecticut	2008	161.2	SEER-17	b	[15, 58]
	Detroit	2008	179.8	SEER-17	b	[15, 58]
	Hawaii	2008	145.9	SEER-17	b	[15, 58]
	Iowa	2008	134.8	SEER-17	b	[15, 58]
	Kentucky	2008	144.7	SEER-17	b	[15, 58]
	Los Angeles	2008	135.3	SEER-17	b	[15, 58]
	Louisiana	2008	161.5	SEER-17	b	[15, 58]
	New Jersey	2008	158.3	SEER-17	b	[15, 58]
	Rural Georgia	2008	173.9	SEER-17	b	[15, 58]
	San Francisco Bay Area	2008	152.2	SEER-17	b	[15, 58]
	San Jose	2008	139.6	SEER-17	b	[15, 58]
	SEER-13	1992	197.0	SEER-13	b	[18, 58]
	SEER-13	1993	213.1	SEER-13	b	[18, 58]
	SEER-13	1994	195.6	SEER-13	b	[18, 58]
	SEER-13	1995	182.9	SEER-13	b	[18, 58]
	SEER-13	1996	182.3	SEER-13	b	[18, 58]
	SEER-13	1997	184.2	SEER-13	b	[18, 58]
	SEER-13	1998	187.3	SEER-13	b	[18, 58]
	SEER-13	1999	194.3	SEER-13	b	[18, 58]
	SEER-13	2000	196.4	SEER-13	b	[1, 13]
	SEER-13	2001	186.0	SEER-13	b	[1, 13]

Region	Location	Year(s)	Age-Standardized Incidence per 100,000	Source	Population of African Descent (%)[a]	Reference
	SEER-13	2002	191.4	SEER-13	b	[1, 13]
	SEER-13	2003	174.7	SEER-13	b	[1, 13]
	SEER-13	2004	171.6	SEER-13	b	[1, 13]
	SEER-13	2005	163.4	SEER-13	b	[1, 13]
	SEER-13	2006	165.7	SEER-13	b	[1, 13]
	SEER-13	2007	175.6	SEER-13	b	[1, 13]
	SEER-13	2008	163.8	SEER-13	b	[1, 13]

[a] Includes men defined as African descent, including those defined as having some non-African ancestry, including creole, mestizo, mulatto and related groups. Percentages denoted as "greater than" indicate that the non-African percentages include African or mixed-African background individuals. NA: Data not available. Source: *The World Factbook 2009*. Washington, DC: Central Intelligence Agency, 2009.

[b] Data refer only to African descent population

Supplementary Table 2: CaP Incidence Data in Men of African Descent Outside the US Derived from the Literature that were Excluded From Consideration Due to Lack of Sampling Frame, Unclear Reference Population, Lack of Age-Standardization, or Other Data Limitations.

Region	Location	Year(s)	Adjusted Incidence per 100,000	Source	Age-Standardization	Reference
Africa	East Africa	1975	5.5	Population	Crude	[59]
	Central Africa	1975	6.0	Population	Crude	[59]
	South Africa	1975	8.0	Population	Crude	[59]
	West Africa	1975	3.0	Population	Crude	[59]
	Congo (Brazzaville)	1996-1999	6.4	Hospital	World	[60]
	Cameroon	1986-1990	93.8	Hospital	Crude	[61]
	Gambia	1997-1998	4.7	Registry	World	[62]
	Gambia	1988-1997	2.5	Registry	World	[62]
	Guinea-Conakry	1992-1994	8.1	Registry	World	[63]
	Guinea-Conakry	1996-1999	9.7	Registry	World	[63]
	Ivory Coast (Abidjan)	1995-1997	31.4	Registry	World	[64]
	Kenya (Eldoret)	1998-2000	16.8	Registry	World	[60]
	Mali (Bamako)	1987-1988	4.7	Registry	World	[65]
	Malawi (Blantyre)	1994-1998	5.9	Registry	World	[66]
	Malawi (Blantyre)	2000-2001	10.7	Registry	World	[66]
	Namibia	1995-1998	21.8	Registry	World	[60]
	Niger (Niamey)	1993-1999	10.8	Registry	World	[60]
	Nigeria (Midwestern)	2008-2009	7.9	Registry	World	[67]
	Nigeria (Southwestern)	2002-2004	182.5	Hospital	Crude	[68]
	Nigeria	1984-1994	61.3	Hospital	Crude	[69]
	Nigeria	1988-1993	127.0	Hospital	Crude	[6]
	Nigeria (Ibadan)	1998-1999	19.8	Registry	World	[60]
	Nigeria (Port Harcourt)	1985-1998	114.0	Hospital	Crude	[70]
	South Africa	1988	14.4	Registry	World	[71]
	South Africa	1989-1992	14.3	Registry	World	[60]
	South Africa (Eastern Cape)	1998-2002	4.4	Registry	World	[72]
	Swaziland	1996-1999	21.5	Registry	World	[60]
	Transkei Umtata	1996-1998	3.7	Registry	World	[60]
	Uganda (Kyadondo)	1960-1966	3.1	Registry	Crude	[55]
	Uganda (Kyadondo)	1967-1971	6.8	Registry	Crude	[55]
Caribbean	Bahamas	2009	228.0	Registry	Bahamas	
	Bermuda	2000-2003	325.1	Registry	USA	[73]
	Caribbean	1975	19.0	Population	Crude	[59]
	Guadeloupe	1995	92.5	Pathology	World	[74]
	Guadeloupe	1996	93.3	Pathology	World	[74]
	Guadeloupe	1997	91.3	Pathology	World	[74]
	Guadeloupe	1998	91.3	Pathology	World	[74]
	Guadeloupe	1999	88.8	Pathology	World	[74]

Region	Location	Year(s)	Adjusted Incidence per 100,000	Source	Age-Standardization	Reference
	Guadeloupe	2000	100.9	Pathology	World	[74]
	Guadeloupe	2001	107.9	Pathology	World	[74]
	Guadeloupe	2002	152.3	Pathology	World	[74]
	Jamaica (Kingston and St. Andrew)	1995	56.4	Registry	World	[75]
	Jamaica (Kingston)	1989-1993	304.0	Registry/ Clinic	USA	[76]
UK	UK-Caribbean	1995-2001	165.5	Clinical Cohort	Europe	[77]
	UK-African	1995-2001	139.3	Clinical Cohort	Europe	[77]
	UK	1999-2000	647.0	Medical Databases, Regional Registry	Europe	[78]
